# A multi-centre quality improvement project to reduce the incidence of obstetric anal sphincter injury (OASI): study protocol

**DOI:** 10.1186/s12884-018-1965-0

**Published:** 2018-08-13

**Authors:** Posy Bidwell, Ranee Thakar, Nick Sevdalis, Louise Silverton, Vivienne Novis, Alexandra Hellyer, Megan Kelsey, Jan van der Meulen, Ipek Gurol-Urganci

**Affiliations:** 1 Royal College of Obstetricians and Gynaecologists, London, UK; 2 Croydon Health Services NHS Trust, Croydon, UK; 3 Health Service & Population Research Department, King’s College London, London, UK; 4 Royal College of Midwives, London, UK; 5 London School of Hygiene & Tropical Medicine, London, UK

**Keywords:** Obstetric anal sphincter injury, OASI, Third degree tears, Fourth degree tears, Perineal trauma, Quality improvement, Care bundle, Childbirth

## Abstract

**Background:**

Third and fourth degree perineal tears, or obstetric anal sphincter injuries (OASI), sustained during childbirth can result in anal incontinence and psychosocial problems which require ongoing treatment. Within the English National Health System (NHS) reported rates of OASI have gradually increased. In response, a care bundle was developed incorporating four elements: 1) antenatal information to women, 2) manual perineal protection during all vaginal births, 3) episiotomy to be performed with a 60° mediolateral angle at crowning (when clinically indicated) and 4) perineal examination (including per rectum) after childbirth. Implementation of the OASI Care Bundle is aided by a skills development module and an awareness campaign. The project is a collaboration between two national professional bodies, an NHS hospital trust and an academic institution.

**Methods:**

Implementation of the OASI Care Bundle will be evaluated using a stepped-wedge design. From January 2017 sixteen maternity units across England, Wales and Scotland will participate in the study over a 15-month period, with sequential roll-out of the intervention in four blocks (regions) of four units. The primary clinical outcome is OASI rate. Regression analysis will adjust for differences in organisational characteristics and obstetric risk factors in women who gave birth before and after implementation of the care bundle. Focus group discussions and in-depth interviews with clinicians will evaluate the feasibility of integrating the care bundle into routine practice. Interviews with women will explore the acceptability of the intervention.

**Discussion:**

This protocol outlines the evaluation of our quality improvement project which aims to prevent OASI using a bundle of evidence-based interventions that are each widely used in practice. The OASI project aims to 1) standardise practice to prevent OASI in a way that is acceptable to clinicians and women and 2) identify the barriers and enablers associated with upscaling interventions within maternity units. If found to be effective, feasible and acceptable, the OASI Care Bundle will be shared with a range of audiences using the communication channels available to the professional bodies.

**Trial registration:**

The OASI Project was retrospectively registered on the ISCTRN12143325 database date assigned 03/10/2017.

## Background

The rates of recorded obstetric anal sphincter injuries (OASI) among primiparous women have tripled in the English National Health Service (NHS) from 1.8% in 2000 to 5.9% by 2011, with 70,000 women being affected during this period [1]. An OASI is defined as any degree of injury to the anal sphincter muscle sustained during childbirth. Short-term consequences of OASI include pain, bleeding and infection, which can result in urinary retention and constipation [2]. Long-term consequences include urinary and faecal incontinence, chronic pain and painful intercourse. Such morbidities can have a severe psychosocial impact and affect future birth choices [3]. Furthermore, there are significant cost implications for the NHS associated with further treatment and negligence claims relating to OASI between 2000 and 2010 are estimated to total £31.2 million [4]. OASI rates are also increasing in several countries including Australia, China and Canada [5–7]. Such trends render OASI prevention a quality improvement priority for maternity units across the world.

The aetiology of OASI is multifaceted and known risk factors include; birthweight greater than 4 kg, persistent occipito-posterior position, primiparity, induction of labour, prolonged second stage and an instrumental (assisted) birth [8, 9]. Research has shown that training gaps amongst midwives and obstetricians and a lack of awareness of risk factors may contribute to the increase in OASI rates [10–16]. Variations in practice, with increasing use of a “hands-poised” approach to protecting the perineum during childbirth as opposed to a “hands-on” approach are also thought to be contributing factors [1]. Not all perineal injuries are preventable, however, evidence from Scandinavia and small-scale studies in England show that OASI rates can be significantly reduced [10, 11, 17–19]. For instance, in Norway, the OASI rate decreased from 4.0 to 1.9% after the introduction of a package of interventions [10].

In light of the rising trends in OASI rates in England, a team of national experts met to discuss strategies to reverse this trend. There was unanimous agreement that there was potential for a ‘care bundle’ of evidence-based actions to be developed. A joint statement supporting this work was produced by the Royal College of Obstetricians and Gynaecologists (RCOG) and the Royal College of Midwives (RCM) [20].

With this in mind, two main objectives were established:

1. To develop and implement a care bundle to reduce third and fourth degree perineal tears
2. Evaluate outcomes associated with implementing a care bundle

We report the development of the OASI Care Bundle; and the design of the prospective evaluation of the care bundle across maternity units in England, Wales and Scotland.

### Development of the OASI care bundle

Care bundles are described by the US Institute for Healthcare Improvement as a collection of interventions that need to be delivered together in order to provide effective and safe care for patients [21]. Within the NHS, care bundles are increasingly being developed to improve outcomes [22]. For a care bundle to be most effective, it should be concise, straightforward and comprise of three to five evidence-based practices [21]. The novelty of the approach is that is combines elements of good practice into one cohesive package that, when implemented, improves the reliability and quality of care, and in turn, patient outcomes.

**Fig. 1:**
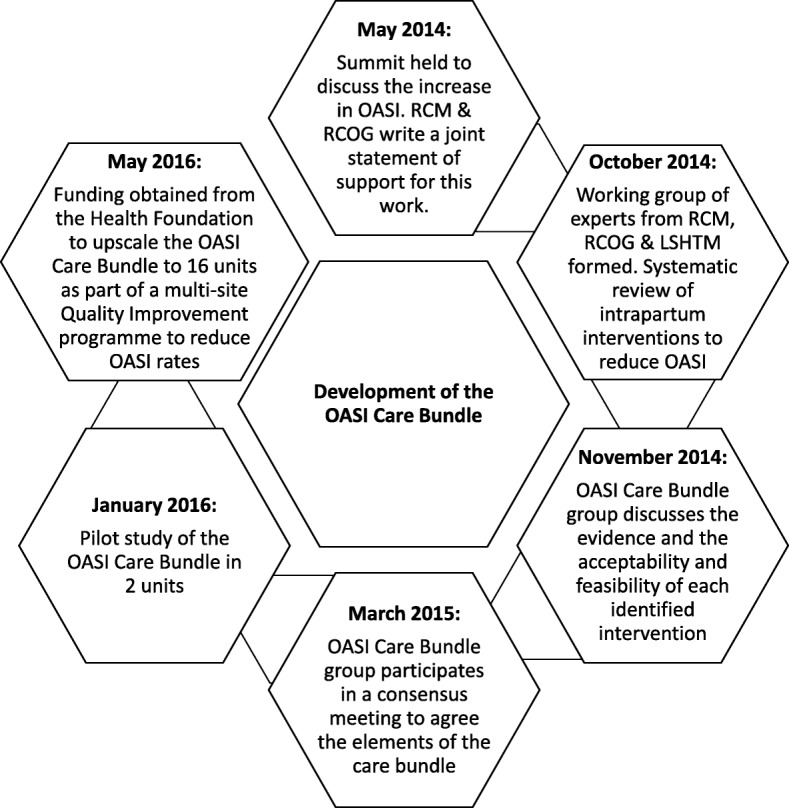
Development of the OASI Care Bundle. This shows the stepwise development of the care bundle which began with the summit of national experts and the formation of a working group (the OASI Care Bundle group) in May 2014

Figure 1 shows the stepwise development of the care bundle which began with a summit of national experts and the formation of a working group (the OASI Care Bundle group) in May 2014. Following this, a literature review of intrapartum interventions that might impact OASI rates was conducted. Over 2000 studies were identified using a comprehensive search strategy within EMBASE, Ovid Medicine, Cochrane, CINAHL and the Maternity and Infant Care database. Studies that were included for review were randomised, or quasi-randomised, trials of interventions that are used in the second stage of labour with OASI as an outcome. Results were supplemented by narrative reviews of key non-randomised studies.

The results of the review and recommendations from pre-existing UK guidelines [9, 23] were presented to the OASI Care Bundle group, comprising of clinical and methodological experts with representatives from the RCOG, RCM and the London School of Hygiene and Tropical Medicine (LSHTM). This led to a structured discussion about the clinical practicalities of each individual care bundle element. The four elements finally included by the OASI Care Bundle group were:

1. Women to be given information during the antenatal period about perineal trauma and how to minimise their risk of sustaining an OASI.
2. When clinically indicated, a mediolateral episiotomy should be performed at a 60-degree angle to the midline at crowning.
3. Documented use of Manual Perineal Protection (MPP) for:
  - All spontaneous vaginal births, unless the woman’s chosen birth position does not allow for it (e.g. water birth)
  - All instrumental vaginal births (e.g. forceps, ventouse and kiwi)
4. Following birth, the perineum should be examined and any tears graded in accordance to RCOG guidelines [9]. The examination should include a per rectum check, even if the perineum appears intact.

Following presentations to the RCM and other leaders of labour care and birth, changes were made to the care bundle manual and the training materials to emphasise support for women’s birth choices, the importance of mobility during labour and natural birth physiology.

In January 2015, a survey was sent to all UK maternity units to assess their interest in piloting the OASI Care Bundle. From the 91 units who responded, two were chosen to carry out a three month pilot study. The main aim of the pilot was to determine whether the care bundle was acceptable and feasible for clinicians. Following the pilot study, funding was obtained from the Health Foundation to adapt and scale-up the OASI Care Bundle as a quality improvement project, to be implemented in sixteen maternity units across England, Wales and Scotland; this forms the current project.

### Implementation of the OASI care bundle

**Fig. 2:**
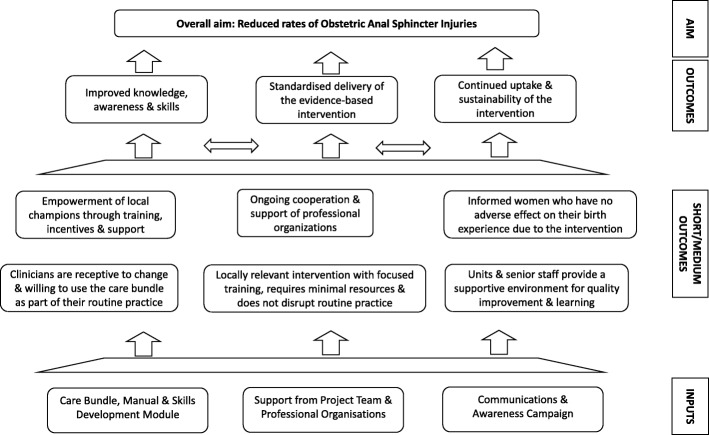
The Theory of Change. This shows our detailed Theory of Change which was developed in conjunction with OASI Care Bundle group

**Fig. 3:**
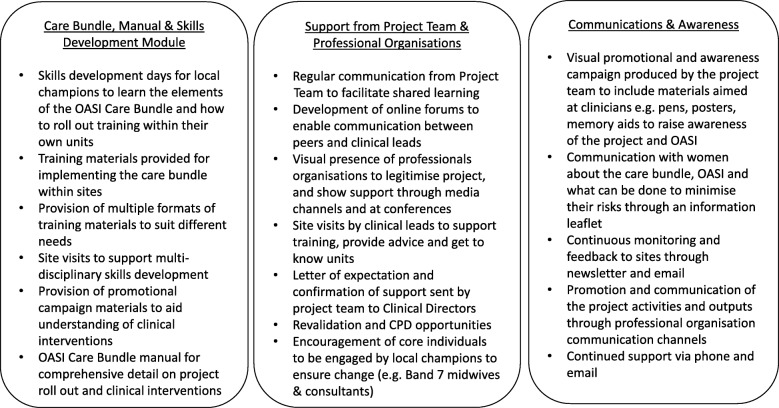
OASI Care Bundle Implementation Optimisation Strategies. This shows the inter-related implementation optimisation strategies for the care bundle

Implementation of the OASI Care Bundle is supported with a skills development module and a communications and awareness campaign. Additionally, sustained leadership and support is provided by professional organisations and the Project Team. This quality improvement project aims to optimise implementation by addressing knowledge gaps regarding the prevention of OASI; in particular inconsistencies in training, skills and practice. Local implementation of the project is led by clinical champions (midwives and obstetricians) within each unit. The champions receive multi-disciplinary training at designated RCOG Skills Development Days (organised and delivered by our Project Team) on the key elements of the care bundle, and they then cascade the training and educational materials to their colleagues in their units. This train-the-trainer approach aims to ensure that all clinicians are trained within the unit within the three month transition phase, thereby achieving adequate enrolment of women to the intervention. Further skills development is provided by the project clinical leads who visit each unit approximately four to six weeks after the start of implementation. A detailed Theory of Change has been developed in conjunction with OASI Care Bundle group (Fig. 2). A number of inter-related implementation optimisation strategies [24] for the bundle have also been articulated (Fig. 3).

### Study design and participants

The overall aim of this quality improvement project is to reduce OASI rates. Secondary outcome measures are acceptability (satisfaction with the intervention), feasibility (extent to which intervention can be applied), coverage (extent to which the population receive the intervention) and sustainability (extent to which intervention is maintained).

#### Study setting

To ensure representation across regions, England, Wales and Scotland were divided into four geographical blocks as follows:

- Region 1: London, South East Coast, Wessex, Thames Valley
- Region 2: Cheshire and Mersey, South West, West Midlands, Wales
- Region 3: East Midlands, East of England, Yorkshire and the Humber
- Region 4: Greater Manchester, North, Scotland

The 91 units which had expressed an interest in taking part in the pilot study were used for the selection process for the scale-up. Within each region, four units were selected to obtain a total of sixteen units, aiming for at least one from each of the clinical senates in the region, at least one from each of the four maternity unit size categories (defined by the National and Perinatal Audit as < 2500, 2500–3999, 4000–5999 and 6000+ births per year [25]) and at least one from each of the three types of maternity units; namely obstetric-led (hospital based care where obstetricians take responsibility for high-risk women and midwives take responsibility for low-risk women), alongside midwifery units (where midwives provide care for low-risk women in co-located units in the same building as an obstetric unit) and freestanding midwifery unit (often called ‘birth centres’, these are geographically separate from obstetric units and midwives provide care for low-risk women) [26]. A list of the sixteen study units can be found on the ISRCTN database.

All vaginal births are potentially eligible for the intervention when the attending clinician (midwife or obstetrician) has been trained to use the care bundle. Women are excluded if they are in a birthing position that makes it impossible to implement all aspects of the care bundle (e.g. water birth). Given an average number of 304 deliveries per month in each unit, a target of 70% of clinicians having been trained (i.e. deliveries being eligible) and the assumption of up to 10% vaginal deliveries being excluded due to birthing positions, the estimated number of women potentially eligible to receive the care bundle intervention is 32,800 (Table 1).

**Table 1:** Design of the stepped wedge trial for OASI Care Bundle evaluation

Region	Time					
	0	1	2	3	4	5
1		Roll-out	3280	3280	3280	3280
2			Roll-out	3280	3280	3280
3				Roll-out	3280	3280
4					Roll-out	3280

## Methods

The study has a stepped-wedge design with complete, continuous recruitment [27]. In stepped-wedge designs, the short-exposure intervention is implemented at the healthcare provider level and staggered in blocks.

The four regions were sequentially randomised to the intervention, using a random number generator. The first region was initiated in January 2017, and the intervention was introduced to remaining regions every three months. The units were informed of their allocation two months before the start of their roll-out period in order to allow for preparation time. The first three months of the intervention is the ‘transition phase’, which is when the care bundle launched at the units, and the local champions cascade the skills development module to their colleagues.

Mixed methods will be used to assess the clinical effectiveness and the implementation outcomes of the OASI Care Bundle. Clinical outcomes will be assessed via routinely collected patient-level data, captured through each unit’s Maternity Information System (MIS). Implementation outcomes will be assessed through specific qualitative and quantitative methods, which will provide a detailed evaluation of the acceptability, feasibility, coverage and sustainability of the intervention.

### Assessing clinical outcomes

The primary clinical outcome measure is the OASI rate which will be evaluated using patient-level data from the unit’s Maternity Information System (MIS).

#### Data source

Patient-level data will be extracted from local electronic MIS for 15 units in England, Wales and Scotland, and from the Scottish Morbidity Record 02 (SMR02) and Scottish Birth Record (SBR) for one unit in Scotland. MIS capture detailed demographic and clinical information related to maternity care and outcomes, with the data entered by midwives and support staff in the antenatal clinic or labour ward. Although the participating units use nine different systems and the data format could slightly differ, there is sufficient similarity between them to develop a single dataset for analysis. SMR02, submitted by maternity units to the Information Services Division Scotland since 1975, collects data for all women admitted as inpatients or day cases to Scottish maternity units [28]. This register is subjected to regular quality assurance checks and since the late 1970s has been more than 99% complete.

The pre-defined data specification for MIS, SMR02 and SBR includes perineal trauma, maternal characteristics (e.g. age, body mass index, parity) and intrapartum care (e.g. episiotomy, induction of labour, epidural use, shoulder dystocia and mode of birth). An 18-month extract of patient-level data (which will include a baseline, transition and implementation phase) will be extracted from the data sources. This will be obtained at the end of March or April 2018 depending on when the region began roll-out to ensure 18 months of data for all units. While data for a longer period for pre-rollout is available, we will follow the recommendation that primary analyses need to be based mainly on data from those exposed to the intervention or control while clusters are in both conditions, supplemented only by data from immediately before or after the roll-out period [29]. The dataset will not include any patient identifiable information. It will be transferred to and stored at a secure server, and only named individuals from the Project Team will have access to the dataset. All participating units have signed a Data Sharing Agreement with the RCOG, and all users of the data are obliged to fully comply with Data Protection Legislation.

#### Data management

Prior to analyses, the data from each unit will be cleaned and re-coded to ensure consistent definitions for all variables. For example, in some units’ MIS, the perineal trauma variable might also include degree of tears (e.g. 3a, 3b, 3c, 4) which will be coded as 1 if the perineal tear is either 3rd or 4th degree.

Data quality will be assessed by checking data completeness, plausible distributions and internal consistency. Data completeness is expected to be very high in the key data items such as birth trauma, mode of birth and birth weight, however, the completeness of other variables such as labour onset and epidural use might be variable across units [25]. Multiple imputation methods will be used to deal with missing data, if possible, following an assessment of the extent and patterns of missing data. Plausible distribution checks include assessing whether the distributions calculated from non-missing data is within a clinically possible and acceptable range (e.g. OASI rates less than 15%). Internal consistency checks include assessing agreement of data that might be present in more than one data field (e.g. repair of a tear would only be recorded in women who had an OASI; or women who delivered vaginally could not have an onset of labour recorded as pre-labour caesarean). Any implausible distributions or high levels of internal inconsistency would highlight data extraction errors or systematic errors in coding, which would need to be discussed with the unit’s MIS team, and a new revised extract will be requested if required.

#### Data analysis

In stepped-wedge designs, power calculations need to take into account both the clustered nature of the design and the confounding effect of time [30]. The statistical power for the study was calculated using the “steppedwedge” command in STATA 14 [31]. The “steppedwedge” command, written by Hemming and Girling [32] and based on the approach proposed by Hussey and Hughes [33], allows for power and detectable-difference calculations for binary outcomes, following the specification of the number of clusters (units) at each step, the number of steps, the average cluster (unit) size, and the intra-cluster correlation coefficient (ICC, rho). In this study, the number of steps and the number of units per step are both four, totalling 16 units. Unit sizes (the number of vaginal deliveries in each participating unit) were available from national maternity statistics data for each country [28, 34, 35]. There were on average 304 vaginal births a month for each participating unit (with a range of 113–539) in 2014/2015, therefore the average cluster size was 912 vaginal deliveries per unit in a three-month period (duration of each step). The baseline OASI rate and the intra-cluster correlation (ICC, rho) were calculated from English maternity clinical indicators data for 2013/14 [36]. For the 13 participating English units, the OASI rate for all vaginal deliveries was 3.2% and the ICC was 0.0062 (CI:0.0018 to 0.0214). The statistical power of the study to detect a 25% reduction in OASI rate (from 3.2 to 2.4%) is 0.92. Under various scenarios (e.g. using lower confidence interval value for ICC, a cluster size of 700 or a baseline OASI rate of 2.8%), the statistical power remains over 80%.

In stepped-wedge studies, the proportion of units exposed to the intervention gradually increases, meaning that unexposed observations will, on average, be from an earlier calendar time than exposed observations [30]. Furthermore, improved reporting and increased awareness of the care bundle over the period is likely to have an impact on the OASI rates [1]. Thus, calendar time is associated with both the exposure to the intervention and also the outcome, and is therefore a potential confounder. We will use logistic mixed effects regression to model the log odds of sustaining an OASI, with a fixed effect for each step and a random effect to account for clustering at the unit level [33, 37]. The model will include a linear secular trend and also adjust for risk factors for OASI (maternal age, BMI, ethnicity, mode of delivery, episiotomy, birthweight, prolonged labour, and shoulder dystocia) [1].

Additional analyses are planned subject to completeness and quality of data collected for monitoring purposes throughout the study, including data on care bundle compliance, either on a paper form or electronically. In addition, these summary measures of organisational readiness and trends in the uptake of the intervention (i.e. percentage of clinicians trained and compliance rate) may also be used to explore the impact of these organisational factors on clinical outcomes.

### Data collection procedures to measure implementation outcomes

**Fig. 4:**
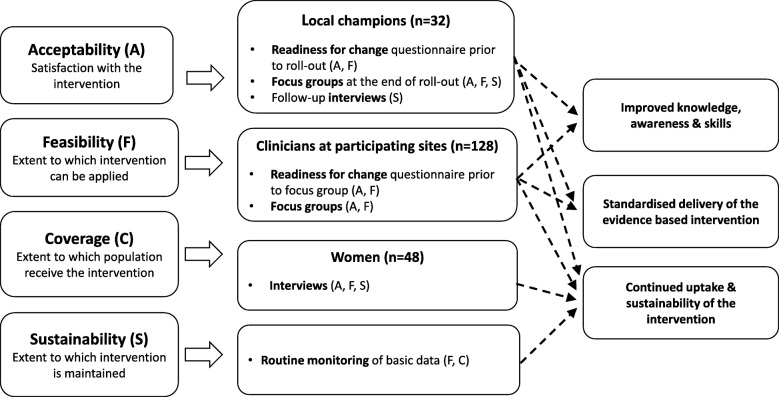
Data collection tools used to evaluate implementation outcomes. This brings together all the process metrics and indicates which implementation outcome they will assess

**Fig. 5:**
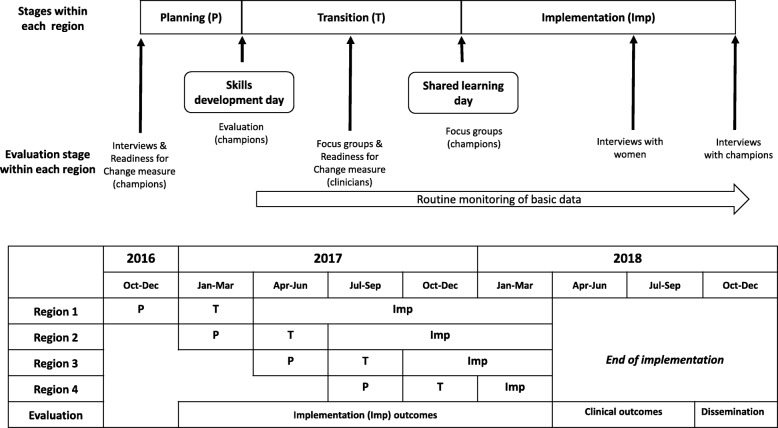
Evaluation timeline outlining implementation components. This illustrates the timing of data collection for each region

The study will prospectively measure the four implementation outcomes (acceptability, feasibility, coverage and sustainability) using quantitative and qualitative methods. Figure 4 brings together all the process metrics and indicates which implementation outcome they will assess and Fig. 5 illustrates the timing of data collection for each region.

#### Organisational change

The first stage of the evaluation will assess readiness for change within each unit. Weiner’s theory of organisational change will be used to measure organisational readiness and change efficacy [38]. This survey, completed by local champions as well as clinicians taking part in focus groups in their units, will assess whether the units have a supportive environment for quality improvement and learning.

#### Focus group discussions (FDGs)

Sixteen FDGs will be conducted with clinicians from participating units (one FDG per unit) during the transition phase. The FDGs will explore whether clinicians are receptive to change and willing to use the OASI Care Bundle as part of their routine practice. Participants will be recruited from clinicians working within the participating units and will comprise a mixture of midwives and obstetricians of varying experiences. Four FDGs will be also conducted with the local champions (one per implementing region) at the end of the transition phase in order to assess whether the care bundle is a locally appropriate intervention that does not interrupt practice.

Standard FDGs procedures will be followed – an experienced moderator will facilitate the session. The FDGs will explore 1) attitudes towards the care bundle, 2) experience of using the care bundle, 3) opinions of the skills development module, the practicalities of how training was rolled out and whether the ‘train the trainer’ model is appropriate, 4) views of the awareness campaign, 5) knowledge of the morbidity associated with perineal trauma, 6) perceptions of contextual factors (e.g. organisational culture, resources, leadership, support and motivational environment) which impact behavioural change interventions. An open-ended topic guide was developed by the evaluation team and clinical leads to facilitate discussion and ensure engagement, exploration and the opportunity for reflections on the care bundle. The information generated from the FGDs will be used to understand acceptability, feasibility, coverage and sustainability of the care bundle. The information will also provide an understanding of the barriers and enablers that are associated with implementing quality improvement within maternity units.

#### In-depth interviews with the local champions

We will conduct in-depth interviews with all of the primary champions at the end of the implementation period. This will mean a total of 32 interviews. The objective of these interviews will explore whether the care bundle has become part of routine practice and whether all elements are being used consistently. Additionally, these interviews will explore the challenges experienced during implementation of the care bundle, feelings of empowerment and support received from the professional organisations.

#### In-depth interviews with women

Current environment encourages informed choice and therefore women’s opinion must be included, where possible, in order to facilitate this. It is suggested that a ‘hands-on’ approach restricts women’s choices and affects their birthing position [39]. A missing element in this continued debate is discussion with women and this is essential to inform evidence-based practice. As mobility in labour and perceptions of pain will be explored during the interviews, for women to be eligible to take part in the interviews, they must have had a vaginal birth without an epidural, or spinal anaesthesia and received the OASI Care Bundle as part of their care. The attending midwife will provide eligible women with information about the interviews and if the woman is interested in taking part, she will be contacted by a member of the OASI Project Team at a convenient time in order to conduct a telephone interview. A proportionate approach to consent will be taken, i.e. by providing contact details and agreeing to a time for the interview, the woman has consented to participating. Women who take part in the interviews are given assurances that everything they say is anonymous, that they are free to withdraw at any time and that taking part will not affect the care that they, or their baby receives. A total of forty-eight interviews will be conducted (three per participating unit). The interviews will explore the woman’s birth story, in particular memories of pain, mobility during labour, birthing position and any guidance that was received during childbirth.

#### Monitoring of clinical data during implementation of the OASI care bundle

Clinical data will be monitored on a weekly basis by the evaluation team. These data include rates of OASI, episiotomy and caesarean sections, as well as the number of eligible and care bundle compliant births. In addition to providing information on coverage, routine monitoring of data may be used to trigger additional Project Team communication or support if an unusual clinical activity is observed. Additionally, evidence of integration of the care bundle into routine practice will be continuously monitored by assessing the number of eligible and care bundle compliant births each week. Statistical process control techniques will identify any unusual patterns in OASI or compliance rates.

Each unit will be sent a six month summary of their OASI rates and compliance data. In order to facilitate shared learning, this six month summary will also be aggregated by region (with anonymization of data) and be provided to the four units within that region. This is a quality improvement project which aims to reduce rates of OASI. With this in mind, a critical review after six months, with sharing of anonymised results amongst neighbouring units, will facilitate learning for all units in order to understand what has gone well and which areas need to be targeted to further accelerate any change in OASI rates. Similar summaries (to individual units and at regional level) will be provided at nine months and twelve months. Provision of such feedback contributes to the ‘Communications and Awareness Campaign’ component of the ‘Theory of Change’ and will provide information about the effectiveness of the care bundle. Such information will promote engagement with the care bundle and help enable change.

Fidelity is seen as a crucial factor to determine whether the care bundle is implemented as it is intended. However, as the care bundle is implemented at time of birth, it is not possible in practice for an impartial observer to monitor whether each care bundle component is correctly carried out for each birth. Where possible, the Project Team advocates the presence of a second midwife at birth who can observe practice and help identify any training needs. Additionally, the awareness campaign, continued skills development, presence of the local champions and unit visits by the Project Team will contribute to ensuring that intervention is correctly implemented.

An independent Advisory Group containing professional stakeholders with relevant expertise in perineal trauma and quality improvement was established. The Advisory Group also contains lay representation to ensure that woman-centred care remains at the fore-front. The Advisory Group provides an independent oversight of project activities and meets with the Project Team every six months.

#### Qualitative data management and analysis

All FDGs and interviews will be audio-recorded. These will be transcribed verbatim and field notes summarized. All interviewees will be assigned a pseudonym. Qualitative data will be analysed during data collection. This interactive and cyclical approach allows thought on existing data in order to generate strategies for collecting new and better quality data [40]. Grounded theory will be used as it allows for hypotheses to be developed using an inductive process [41]. Progressive coding systems allow for use of existing theory as an orientating device, whilst maintaining an openness to new concepts. After codes have been developed they will be applied to the data using NVivo. ‘Axial coding’ will determine causal or consequential relationships between the codes, in order to identify any dominant themes. Finally, selective coding, will be used to illustrate the themes obtained in axial and open coding in order to produce quotable material for the results [40].

A simple manifest analysis of the qualitative material [42] will be conducted, as we are interested in documenting what clinical practice is occurring and clinicians’ experiences. Data will be analysed both deductively and inductively [43]. Interview transcripts and observation narratives will be coded thematically.

Triangulation of these different strands of data will provide in-depth information about the barriers and enablers associated with uptake and scaling up of the intervention and assess the outcomes identified in the theory of change (Fig. 2), namely: improved knowledge, awareness and skills; and standardised, continuous and sustainable delivery of the intervention.

### Dissemination plan

The quantitative analysis will commence once all the MIS data has been submitted by the units (May 2018). The qualitative analysis will begin once data collection is completed for each evaluation component – FGDs with clinicians (December 2017), FGDs with champions (January 2018), interviews with women (March 2018) and interviews with champions (April 2018). Results will be reviewed by the Project’s Advisory Group prior to release.

All results will ready for the OASI Care Bundle dissemination event at the RCOG in November 2018. The event will be open to clinicians (doctors and midwives), patient and public representatives, policy makers and commissioners from around the country to ensure wide audience is captured. High-level aggregate data will be presented, individual units will not be identifiable. Participating units will each be given an individualised summary of their data.

Results will also be submitted for publication in peer reviewed publications and the final evaluation report is due to be submitted to the Health Foundation in January 2019. Through the national channels of the RCOG and the RCM, the report will be shared with a range of audiences (e.g. patient and public, commissioners, clinicians, and policy makers) to ensure a wide dissemination. A lay summary will be provided within the report to ensure it is available to both clinicians and the patient and public.

Attendance at conferences will take place during the study and after completion and presentations will focus on the appropriate stage of the project. All publication will acknowledge the Health Foundation, study units and participants.

## Discussion

The morbidities associated with OASI can severely impact a woman’s quality of life. There is a belief among some NHS clinicians that OASIs are an unavoidable consequence of vaginal birth and this project challenges that conventional and harmful view.

The strengths of this study are that a stepped-wedge design has been used to assess the impact of the OASI Care Bundle. A multi-methods evaluation will assess feasibility, acceptability, coverage and sustainability. Evaluation of the OASI Care Bundle is in partnership with LSHTM, who are methodological experts, with a strong background in generating quantitative evidence on the processes and outcomes of obstetric care, as well as its determinants.

Leadership for the OASI Care Bundle is provided by both the RCM and the RCOG. This high-profile endorsement has provided unity amongst clinicians. The project receives significant input from lay members within the governance structure and from women, both individually and collectively, through the RCOG’s Women’s Network. This has ensured that woman-centred care remains the focus.

This project aims for a standardised and acceptable practice for the prevention of OASI. The novelty of the OASI Care Bundle is that it combines good practice into one cohesive bundle and is supported by a skills development module, as well as an awareness campaign.

## Abbreviations

FGD: Focus Group Discussion
LSHTM: London School of Hygiene and Tropical Medicine
MIS: Maternity Information System(s)
MPP: Manual Perineal Protection
NHS: National Health Service, UK
OASI: Obstetric Anal Sphincter Injury
RCM: Royal College of Midwives
RCOG: Royal College of Obstetricians and Gynaecologists
